# Efficacy and safety of 25 and 50 μg desmopressin orally disintegrating tablets in Japanese patients with nocturia due to nocturnal polyuria: Results from two phase 3 studies of a multicenter randomized double‐blind placebo‐controlled parallel‐group development program

**DOI:** 10.1111/luts.12276

**Published:** 2019-08-09

**Authors:** Osamu Yamaguchi, Kristian V. Juul, Ali Falahati, Toru Yoshimura, Futoshi Imura, Mikiya Kitamura

**Affiliations:** ^1^ Division of Lower Urinary Tract Symptom Research Nihon University School of Engineering Koriyama Japan; ^2^ Ferring International Pharmascience Center Copenhagen Denmark; ^3^ Biometrics, R&D Ferring Pharmaceuticals Co. Ltd Tokyo Japan; ^4^ Project Development, R&D Ferring Pharmaceuticals Co. Ltd Tokyo Japan

**Keywords:** desmopressin, nocturia, nocturnal polyuria

## Abstract

This study assessed the efficacy and safety of desmopressin orally disintegrating tablets (ODTs) in Japanese males (50 and 25 μg) and females (25 μg) with nocturia due to nocturnal polyuria (NP). Two Phase 3 randomized double‐blind placebo‐controlled studies of 342 males and 190 females with nocturia due to NP were conducted. The primary endpoint was change from baseline in mean number of nocturnal voids. In addition, time to first awakening to void, nocturnal urine volume, NP index (NPI), and quality of life were assessed during a 12‐week treatment period. In males, 50 and 25 μg desmopressin ODTs significantly reduced the number of nocturnal voids by −1.21 (*P* < .0001) and − 0.96 (*P* = .0143), respectively, and significantly prolonged the time to first awakening to void by 117.60 minutes (*P* < .0001) and 93.37 minute (*P* = .0009), respectively, with no safety concerns. In females, 25 μg desmopressin ODT significantly prolonged the time to first awakening to void by 116.11 minutes (*P* = .0257), with no safety concerns. The reduction in the number of nocturnal voids (−1.11) was not significantly different compared with placebo (*P* = .0975). Desmopressin ODTs (50 and 25 μg) were an effective and well‐tolerated treatment for nocturia due to NP in Japanese males, and desmopressin ODT 50 μg is an appropriate dose in these patients. For patients who are likely to experience hyponatremia, such as elderly males, starting with 25 μg desmopressin ODT should be considered.

## 1 INTRODUCTION

Nocturia is a lower urinary tract symptom that occurs more frequently in both males and females with aging. It was defined as “Waking to pass urine during the main sleep period” by the International Continence Society (ICS) in 2018.^1^, ^2^ Nocturia is thought to be primarily caused by nocturnal polyuria (NP), which is the excretion of an excessive amount of urine while sleeping despite 24‐hour urine volume being normal. However, the ICS Standardisation Committee defined nocturia more specifically as nocturnal urine volume that exceeds 33% of the total 24‐hour urine volume for elderly patients, and 20% for younger patients.^2^ Repeatedly waking up at night to void due to NP causes lack of sleep and reduced sleep quality, which markedly reduces a patient's quality of life (QoL), interfering with daily life. Therefore, appropriate treatment is needed. Nocturia associated with NP has been reported to be related to decreased secretion of the antidiuretic hormone arginine vasopressin (AVP).^3^

Desmopressin acetate, a synthetic AVP analog, has proven to be an effective and well‐tolerated treatment in both male^4^ and female^5^ patients with nocturia. An orally disintegrating tablet (ODT) containing desmopressin quickly disintegrates when placed under the tongue and can be taken without water, simplifying administration.

At the present time, 60, 120, and 240 μg desmopressin ODTs (daily doses of 60, 120 and 240 μg, respectively) and 100 and 200 μg desmopressin tablets (daily doses of 100, 200 and 400 μg) have been approved in over 80 countries worldwide for the indication “nocturia associated with idiopathic NP in adults”, and 25 μg desmopressin ODT for women and 50 μg desmopressin ODT for men have been approved in over 35 countries worldwide that can be used more safely, especially by patients who are likely to experience hyponatremia. The efficacy of desmopressin ODT 25 μg desmopressin ODT for women and 50 μg desmopressin ODT for men was confirmed as statistically superior to placebo in all efficacy endpoints in global studies.^6^, ^7^

In Japan, a Phase 2 study suggested a lower dose of desmopressin ODT may be effective in Japanese male and female patients with nocturia.^8^ The study also confirmed a sex difference in sensitivity to desmopressin, and that the optimum dose for NP treatment is lower in females than in males.^8^ Herein we report the results of two separate clinical studies (NCT02904759 and NCT02905682) of the efficacy and safety of 25 and 50 μg desmopressin ODTs in male patients and 25 μg desmopressin ODT in female patients versus placebo. The primary efficacy endpoint was change from baseline in the mean number of nocturnal voids during 12 weeks of treatment. Other secondary endpoints, namely time to first awakening to void, nocturnal urine volume, NP index (NPI), and patient‐reported outcomes, and safety were also evaluated.

## 2 METHODS

### 2.1 Study design

The clinical program comprised two separate clinical studies in male and female patients with nocturia (two or more nocturnal voids) due to NP defined as an NPI ≥33%. During the 12‐week treatment period randomized double‐blind parallel group studies, desmopressin was compared to placebo at 57 (NCT02904759) and 50 (NCT02905682) sites in Japan from September 2016 to September 2017. The studies were approved by the institutional review board for each site and were conducted in accordance with the Declaration of Helsinki, Good Clinical Practice of International Conference on Harmonisation, Good Clinical Practice in Japan, and applicable regulatory requirements. All patients provided written informed consent before participating in the studies.

### 2.2 Patients

For screening, eligible patients in both studies were male or female aged ≥20 years with at least two nocturnal voids for 6 months or more. Prior to the randomization, a 1‐week placebo run‐in/lifestyle change was conducted. During this period, patients were instructed to follow the water consumption and voiding guidance. Subsequently, the eligibility criteria were two or more nocturnal voids (average of 3 days); NP, defined as an NPI ≥33%; and bothered by nocturia, defined as a score ≥ 2 on the Hsu five‐point Likert bother scale (moderate to extremely bothered).^9^

Patients with interstitial cystitis (IC), overactive bladder (OAB), or benign prostatic hyperplasia (BPH) exceeding the stipulated criteria, urinary disorders (eg, severe stress incontinence, heart disease, and uncontrolled hypertension or diabetes) were excluded to eliminate the effects of these conditions on the efficacy evaluation of desmopressin ODT for nocturia associated with NP. Patients with coexisting psychogenic or habitual polydipsia (24‐hour urine output >2.8 L), hyponatremia (serum sodium concentration < 135 mEq/L), or syndrome of inappropriate antidiuretic hormone secretion (SIADH) were excluded to avoid the risk of onset of hyponatremia.

Sample size was based on the hypothesis of demonstrating superiority of 25 μg desmopressin ODT in female patients and superiority of 50 and 25 μg desmopressin ODTs in male patients when tested against placebo on the primary endpoint, with an overall power of 90% and an overall Type I error rate of 5% (two‐sided). Therefore, 89 patients for each group in the female study and 100 patients for each group in the male study were required to demonstrate superiority of desmopressin ODT.

### 2.3 Study treatment and procedures

The studies were comprised of placebo run‐in/lifestyle changes and double‐blind periods. During the placebo run‐in/lifestyle changes period, patients took one placebo ODT every night approximately 1 hour prior to bedtime for 1 week. All subjects were given instructions regarding lifestyle changes (eg, limiting fluid intake and emptying the bladder before going to bed) throughout the entire duration of the trial (including the placebo run‐in/lifestyle changes period). Subsequently, patients who satisfied the inclusion and exclusion criteria were randomized to one of two treatment groups (25 μg desmopressin ODT, placebo) in a 1: 1 ratio for females and three treatment groups (50 or 25 μg desmopressin ODT, placebo) in a 1:  1:  1 ratio for males, stratified by age (<65 and ≥65 years).

In the 12‐week double‐blind period, patients took the investigational product every night approximately 1 hour prior to bedtime for 12 weeks. In addition, subjects recorded nocturnal voiding information and sleep status using a voiding diary for three consecutive days immediately before each scheduled visit, and completed the Nocturia Quality‐of‐Life (N‐QoL) questionnaire to assess the effect of nocturia on health‐related QoL.^10^, ^11^, ^12^ The N‐QoL has been translated and culturally validated to a Japanese setting.^13^ The quality of sleep was evaluated using the Insomnia Severity Index (ISI),^14^ and the bothersomeness of nocturia was assessed using the Hsu five‐point Likert bother scale.^9^

To monitor hyponatremia, patients with a serum sodium concentration <135 mM at screening were excluded from the studies, and serum sodium concentrations were measured on Days 3 and Weeks 1, 2, 4, 8 and 12, or post‐dose for discontinued subject. Subjects were asked to come to the hospital for additional tests and examinations if the serum sodium concentration was ≤130 mM. Subjects were instructed to stop taking the investigational product if their serum sodium concentration was ≤125 mM. In addition, subjects were withdrawn from the study if they were diagnosed with water intoxication, if B‐type natriuretic peptide (BNP) exceeded 100 pg/mL, or if 24‐hour urine volume exceeded 2.8 L due to psychogenic or habitual polydipsia during the trial period.

### 2.4 Study endpoints

The primary efficacy endpoint was change from baseline in the mean number of nocturnal voids during 12 weeks of treatment, which has been the standard primary efficacy endpoint in multiple clinical trials of nocturia since overseas clinical trials began in the early 2000s.^15^ The secondary efficacy endpoints were: (a) change from baseline in mean time to first awakening to void (first continuous sleep time) to evaluate the clinical significance as a nocturia medication, (b) change from baseline in mean nocturnal urine volume, which indicates the pharmacological effect of desmopressin ODT, (c) change from baseline in mean NPI, which evaluates the lowering effect on the proportion of urine produced at night, and (d) changes in health‐related QoL, quality of sleep, and level of bother of nocturia.

he safety and tolerability of desmopressin ODT treatment were evaluated by the monitoring of adverse events (AEs) based on hematology, blood chemistry, urinalysis, vital signs, and physical findings. AEs were coded to system organ class (SOC) and preferred term (PT) using Medical Dictionary for Regulatory Activities (MedDRA/J), https://www.meddra.org/how-to-use/support-documentation/japanese.

### 2.5 Statistical analysis

The primary efficacy endpoint was assessed for the full analysis set (FAS), which consisted of all randomized (as planned) patients who had at least one post‐baseline measurement for the mean number of nocturnal voids. Analysis was performed based on the planned (randomly assigned) treatment. Desmopressin ODT groups were compared with the placebo group with regard to change from baseline in efficacy variables using longitudinal analysis by repeated‐measures analysis of covariance (ANCOVA), with baseline value as a covariate and treatment, visit, age group, and interaction between treatment and visit as fixed effects.

In the male study where multiple doses were evaluated, the overall significance level was controlled at 5% with a closed testing procedure where the superiority of the low dose was tested against placebo using a two‐sided test at a 5% significance level after superiority of the high dose was established against placebo using a two‐sided test at a 5% significance level.

## 3 RESULTS

### 3.1 Study population

In the male study, 342 male patients were randomized: 109 and 115 in the 50 and 25 μg desmopressin ODT groups, respectively, and 118 patients to the placebo group. Of the randomized subjects, 338 male patients were included in the FAS because all data from the double registration subjects were excluded from the FAS. Finally, 318 patients (93.0%) completed the study, and 24 (7%) withdrew, primarily because of onset of an AE and withdrawal of consent. In the female study, 190 patients were randomized (92 and 98 in the 25 μg and placebo groups, respectively) and 187 female subjects were included in the FAS. Finally, 185 patients (97.4%) completed the trial, and 5 (2.6%) withdrew primarily because of onset of an AE. The number of subjects who withdrew from the study because of an AE was 4 (3.5%) and 2 (1.8%) in the 25 and 50 μg desmopressin ODT groups in the male study and 2 (2.2%) in the 25 μg desmopressin ODT group in the female study.

Patient demographics and characteristics are given in Table 1. There were no differences of note between the treatment groups with regard to demographic data and baseline voiding parameters in both studies (Table 2). The mean number of nocturnal voids at baseline was similar across treatment groups (2.41‐2.53 voids in males; 2.41‐2.46 in females). The mean time to first void at baseline was 152 to 158 minutes in males and 144 to 150 minutes in females.

**Table 1:** Demographic and baseline characteristics for the full analysis set

	Males			Females	
	Placebo (n = 117	Desmopressin ODT		Placebo (n = 97)	Desmopressin (25 μg) ODT (n = 90)
		25 μg (n = 113)	50 μg (n = 108)		
Mean (±SD) age (y)	63.2 ± 12.0	63.2 ± 12.6	62.9 ± 11.9	58.1 ± 13.6	61.4 ± 11.8
Age category					
< 65 y	56 (47.9)	55 (48.7)	49 (45.4)	58 (59.8)	50 (55.6)
≥ 65 y	61 (52.1)	58 (51.3)	59 (54.6)	39 (40.2)	40 (44.4)
Sex					
Male	117 (100.0)	113 (100.0)	108 (100.0)	0	0
Female	0	0	0	97 (100.0)	90 (100.0)
Mean (±SD) BMI (kg/m^2^)	23.4 ± 2.9	24.0 ± 3.3	23.4 ± 3.1	22.9 ± 4.2	22.1 ± 3.5
Ethnicity					
Not Hispanic or Latino	117 (100.0)	113 (100.0)	108 (100.0)	97 (100.0)	90 (100.0)
Race					
Asian	117 (100.0)	113 (100.0)	108 (100.0)	97 (100.0)	90 (100.0)
CKD stage					
eGFR 61–89 (mL/min)	103 (88.0)	100 (88.5)	101 (93.5)	79 (81.4)	74 (82.2)
eGFR ≥90 (mL/min)	14 (12.0)	13 (11.5)	7 (6.5)	18 (18.6)	16 (17.8)

*Note*: Unless indicated otherwise, data are given as n (%).

Abbreviations: BMI, body mass index; CKD, chronic kidney disease; eGFR, estimated glomerular filtration rate; ODT, orally dissolving tablet.

**Table 2:** Baseline voiding parameters for the full analysis set

	Males			Females	
	Placebo (n = 117)	Desmopressin ODT		Placebo (n = 97)	Desmopressin (25 μg) ODT (n = 90)
		25 μg (n = 113)	50 μg (n = 108)		
Mean no. nocturnal voids	2.41 ± 0.64	2.44 ± 0.65	2.53 ± 0.95	2.41 ± 0.54	2.46 ± 0.59
Mean no. nocturnal voids category					
2	64 (54.7)	55 (48.7)	55 (50.9)	46 (47.4)	41 (45.6)
> 2–3	30 (25.6)	35 (31.0)	29 (26.9)	32 (33.0)	28 (31.1)
≥ 3–4	19 (16.2)	19 (16.8)	16 (14.8)	16 (16.5)	19 (21.1)
≥ 4–5	2 (1.7)	2 (1.8)	5 (4.6)	2 (2.1)	1 (1.1)
≥ 5–6	2 (1.7)	2 (1.8)	1 (0.9)	1 (1.0)	1 (1.1)
≥ 6–7	0	0	1 (0.9)	0	0
≥ 7	0	0	1 (0.9)	0	0
Time to first void (min)	158 ± 55	155 ± 52	152 ± 57	144 ± 50	150 ± 55
Nocturnal urine volume (mL)	668.7 ± 197.2	693.6 ± 197.0	730.2 ± 204.4	707.2 ± 205.5	651.0 ± 177.8
NPI (%)	42.8 ± 7.76	42.6 ± 7.76	42.1 ± 7.70	42.4 ± 7.55	40.5 ± 6.02
Mean NPI category ≥33%	117 (100.0)	113 (100.0)	108 (100.0)	97 (100.0)	90 (100.0)
Maximum 24‐h urine volume (mL)	1818.9 ± 524.6	1854.1 ± 486.8	1949.7 ± 465.3	1819.0 ± 407.1	1765.6 ± 394.9

*Note*: Data are given as the mean ± SD or as n (%).

Abbreviations: NPI, nocturnal polyuria index; ODT, orally dissolving tablet.

### 3.2 Efficacy

Results of the efficacy endpoints are given in Tables 3 and 4.

**Table 3:** Adjusted treatment differences for changes from baseline in efficacy variables (voiding parameters) in males and females (full analysis set)

	Males			Females	
	Placebo (n = 117)	Desmopressin ODT		Placebo (n = 97)	Desmopressin (25 μg) ODT (n = 90)
		25 μg (n = 113)	50 μg (n = 108)		
No. nocturnal voids					
Adjusted mean (no. voids)	−0.76	−0.96	−1.21	−0.95	−1.11
Treatment contrast		−0.20	−0.45		−0.16
95% CI		−0.36, −0.04	−0.61, −0.28		−0.34, 0.03
*P*‐value		0.0143	<0.0001		0.0975
Time to first awakening to void					
Adjusted mean (min)	62.97	93.37	117.60	93.53	116.11
Treatment contrast		30.40	54.63		22.59
95% CI		12.47, 48.34	36.47, 72.80		2.77, 42.40
*P*‐value		0.0009	<0.0001		0.0257
Nocturnal urine volume					
Adjusted mean (mL)	−161.18	−225.79	−267.87	−168.47	−248.95
Treatment contrast		−64.61	−106.68		−80.48
95% CI		−99.61, −29.60	−142.36, −71.01		−118.85, −42.12
*P*‐value		0.0003	<0.0001		<0.0001
NPI					
Adjusted mean (%)	−5.81	−9.46	−12.56	−7.43	−11.67
Treatment contrast		−3.65	−6.75		−4.25
95% CI		−5.51, −1.80	−8.63, −4.87		−6.25, −2.24
*P*‐value		0.0001	<0.0001		<0.0001

Abbreviations: CI, confidence interval; NPI, nocturnal polyuria index; ODT, orally dissolving tablet.

**Table 4:** Adjusted treatment differences for changes from baseline in efficacy variables (health‐related quality of life) in male and female patients (full analysis set)

	Males			Females	
	Placebo	Desmopressin ODT		Placebo	Desmopressin (25 μg) ODT
		25 μg	50 μg		
N‐QoL: Total score					
No. subjects	117	112	105	97	90
Adjusted mean	6.87	7.87	9.80	9.45	10.95
Difference between groups		1.00	2.93		1.50
95% CI		−3.00, 5.01	−1.13, 6.99		−3.51, 6.51
*P*‐value		0.6224	0.1568		0.5565
N‐QoL: Global QoL score					
No. subjects					
Adjusted mean	15.71	18.04	20.49	15.46	22.30
Difference between groups		2.33	4.78		6.85
95% CI		−3.87, 8.53	−1.50, 11.06		−0.70, 14.39
*P*‐value		0.4608	0.1354		0.0751
ISI: Total score					
No. subjects	117	112	105	97	90
Adjusted mean	−2.47	−2.66	−3.15	−2.84	−3.15
Difference between groups		−0.19	−0.68		−0.31
95% CI		−1.28, 0.90	−1.79, 0.42		−1.61, 0.99
*P*‐value		0.7323	0.2253		0.6378
Hsu 5‐point Likert bother scale score					
No. subjects	117	112	105	97	90
Adjusted mean	−0.92	−1.08	−1.23	−1.09	−1.24
Difference between groups		−0.16	−0.32		−0.15
95% CI		−0.40, 0.08	−0.56, −0.07		−0.42, 0.11
*P*‐value		0.1827	0.0113		0.2643

*Note*: As per the statistical analysis plan, the interaction term between Treatment and Visit was removed from the original model because it was not significant at the 5% level in the original model.

Abbreviations: CI, confidence interval; ISI, Insomnia Severity Index; N‐QoL, Nocturia Quality‐of‐Life Questionnaire; ODT, orally dissolving tablet; QoL, quality of life.

#### 3.2.1 Number of nocturnal voids

**Figure 1:**
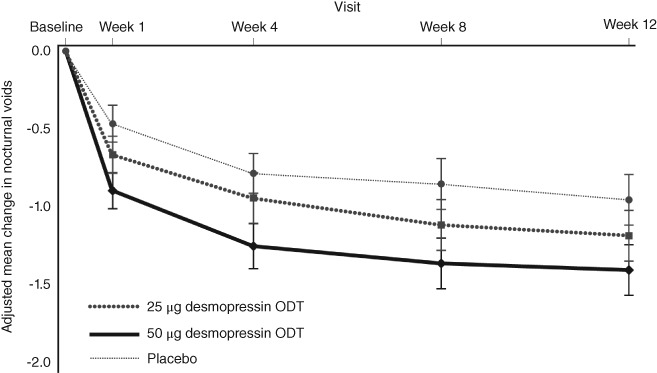
Change from baseline in the mean number of nocturnal voids throughout the 12‐week treatment period in males (full analysis set). Data are the mean ± 95%CI. ODT, orally dissolving tablet

In males, the change from baseline in the mean number of nocturnal voids during 12 weeks of treatment, as a primary endpoint, in the 50 and 25 μg desmopressin ODT and placebo groups was −1.21, −0.96, and − 0.76, respectively. A significant reduction compared with the placebo group was found in both treatment groups (*P* < .0001 and *P* = .0143, respectively), confirming that 50 and 25 μg desmopressin ODTs are effective in the treatment of nocturia in male patients (Table 3; Figure 1). The absolute change from baseline in the 50 μg desmopressin ODT group was 26% larger than that in the 25 μg desmopressin ODT group.

**Figure 2:**
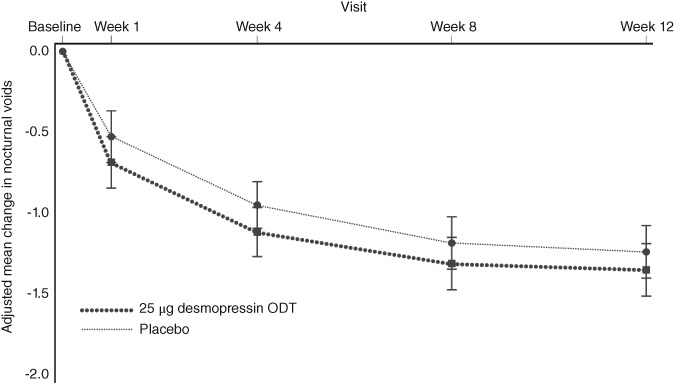
Change from baseline in the mean number of nocturnal voids throughout the 12‐week treatment in females (full analysis set). Data are the mean ± 95%CI. ODT, orally dissolving tablet

In females, the change from baseline in the mean number of nocturnal voids in the 25 μg desmopressin ODT and placebo groups was −1.11 and − 0.95, respectively. No significant reduction compared with the placebo group was observed in the 25 μg desmopressin ODT group, but the reduction was numerically greater in the latter group compared with the placebo group (*P* = .0975; Table 3; Figure 2).

#### 3.2.2 Time to first awakening to void, nocturnal urine volume, and NPI

**Figure 3:**
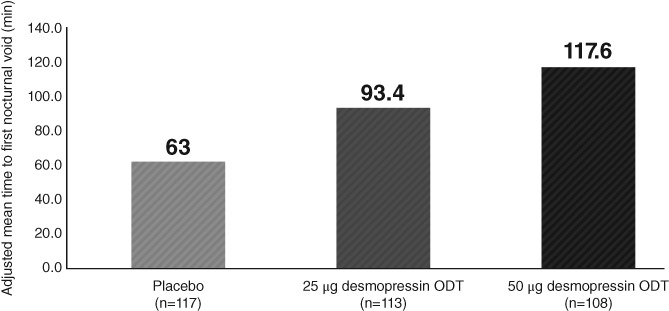
Change from baseline in the mean time to first awakening to void throughout the 12‐week treatment in males (full analysis set). ODT, orally dissolving tablet

In males, the adjusted mean of prolonged time to first awakening to void from baseline in the 50 and 25 μg desmopressin ODT and placebo groups was 117.60, 93.37, and 62.97 minutes, respectively (treatment contrast [TC] vs placebo for the 50 and 25 μg desmopressin ODT groups: 54.63 minutes [*P* < .0001] and 30.40 minutes [*P* = .0009], respectively). In addition, the reduced nocturnal urine volume was 267.87 and 225.79 mL in the 50 and 25 μg desmopressin ODT groups, respectively, compared with 161.18 mL in the placebo group (TC vs placebo for the 50 and 25 μg desmopressin ODT groups: 106.68 mL [*P* < .0001] and 64.61 mL [*P* = .0003], respectively). Furthermore, NPI, which indicates the pharmacological action of desmopressin ODT, showed significant improvements (TC vs placebo for the 50 and 25 μg desmopressin ODT groups: 6.75% [*P* < .0001] and 3.65% [*P* = .0001], respectively). The results of the secondary efficacy endpoints throughout the 12 weeks were supportive of the primary endpoint (Table 3; Figure 3).

**Figure 4:**
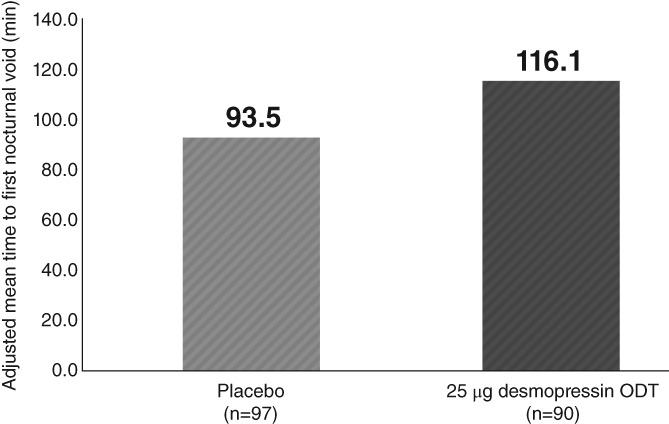
Change from baseline in the mean time to first awakening to void throughout the 12‐week treatment in females (full analysis set). ODT, orally dissolving tablet

Similarly, there was a greater improvement in females, with significant differences in the 25 μg desmopressin ODT group in the time to first awakening to void (TC 22.59 minutes; *P* = .0257), nocturnal urine volume (80.48 mL; *P* < .0001), and NPI (4.25%; *P* < .0001) compared with placebo (Table 3; Figure 4).

#### 3.2.3 Health‐related QoL: N‐QoL, ISI, and Hsu five‐point Likert bother scale

In males in the 50 μg desmopressin ODT group, the change from baseline on the Hsu five‐point Likert bother scale score throughout the 12 weeks was −1.23, indicating a statistically significant improvement compared with placebo (*P* = .0113). The remaining health‐related QoL parameters in the 50 and 25 μg desmopressin ODT groups showed an improvement compared with placebo, although the differences did not reach statistical significance (Table 4). In females, there was a trend towards improvement in health‐related QoL (ie, N‐QoL, ISI, and Hsu five‐point Likert bother scale) with desmopressin ODT treatment versus placebo, but the differences were not significant (Table 4).

### 3.3 Safety and tolerability

The safety results are summarized in Table 5. In males, the frequency of AEs in the 50 and 25 μg desmopressin ODT and placebo groups was 18.3%, 40.0%, and 26.5%, respectively, and the frequency of related AEs (ie, AEs judged by the investigator to have a reasonable possibility of being related to the investigated product) was 5.5%, 7.8%, and 6.0%, respectively. In females, the frequency of AEs in the 25 μg desmopressin ODT and placebo groups was 35.9% and 21.4%, respectively, and the frequency of related AEs was 3.3% and 5.1%, respectively. The most frequent AEs were viral upper respiratory tract infection (4.6%, 4.3%, and 6.8% in the 50 and 25 μg desmopressin ODT and placebo groups, respectively, in males; and 14.1% and 5.1% in the 25 μg desmopressin ODT and placebo groups, respectively, in females). Most AEs were mild or moderate in intensity. There were no deaths or serious related AEs. No detectable trends in the frequency and the characteristics of AEs, except for hyponatremia in both males and females, were confirmed.

**Table 5:** Summary of safety (safety analysis set)

	Males			Females	
	Placebo	Desmopressin ODT		Placebo	Desmopressin (25 μg) ODT
		25 μg	50 μg		
Incidence of AEs	26.5 (31/117)	40.0 (46/115)	18.3 (20/109)	21.4 (21/98)	35.9 (33/92)
Viral upper respiratory tract infection^a^	6.8 (8/117)	4.3 (5/115)	4.6 (5/109)	5.1 (5/98)	14.1 (13/92)
BNP increased^a^	4.3 (5/117)	2.6 (3/115)	0.9 (1/109)	6.1 (6/98)	3.3 (3/92)
Incidence of related AEs^b^	6.0 (7/117)	7.0 (8/115)	5.5 (6/109)	5.1 (5/98)	3.3 (3/92)
BNP increased	0.9 (1/117)	1.7 (2/115)	0.9 (1/109)	2.0 (2/98)	0
Hyponatremia	0	0	1.8 (2/109)	0	0
Constipation	0	0.9 (1/115)	0	1.0 (1/98)	0
Diarrhea	0	0	0	1.0 (1/98)	0
Fatigue	0	0	0	0	1.1 (1/92)
Malaise	0	0.9 (1/115)	0	0	1.1 (1/92)
Blood uric acid decreased	0	0	0	0	1.1 (1/92)
Incidence of SAEs	0	0.9 (1/115)	0.9 (1/109)	0	1.1 (1/92)
Incidence of related SAEs	0	0	0	0	0
Incidence of hyponatremia and blood sodium decreased^c^	0	0	2.8 (3/109)	0	0
Blood sodium decreased	0	0	0.9 (1/109)	0	0
Hyponatraemia	0	0	1.8 (2/109)	0	0
Hyponatraemia and blood sodium decreased					
For age ≥ 65 y	0	0	5.0 (3/60)	0	0
For age < 65 y	0	0	0	0	0
Incidence of significant hyponatremia^d^	0	0.9 (1/115)	1.8 (2/109)	0	0
Incidence of serious hyponatremia^e^	0	0	0	0	0
Incidence of hyponatremia with clinical symptoms	0	0	0	0	0

*Note*: Data are given as percentages (no. subjects with observation/total no. subjects).

Abbreviations: BNP, B‐type natriuretic peptide; SAE, serious AE.

a Treatment‐emergent adverse event (AE) with an incidence ≥2% in any treatment group.

b Related AEs were AEs judged by the investigator to have a reasonable possibility of being related to the investigational medical product; those with an incidence ≥1% in any treatment group are listed in the table.

c Hyponatremia and blood sodium decreased reported as an AE.

d Significant hyponatremia was defined as a serum sodium concentration < 130 mM.

e Serious hyponatremia was defined as a serum sodium concentration ≤ 125 mM.

[Correction added on 22 August 2019, after first online publication: the value under Male Desmopressin ODT's 2nd column has been corrected from ‘25’ to ‘50’ μg.].

Because of the pharmacological effect of desmopressin, hyponatremia was an AE of special interest. According to MedDRA PT, hyponatremia could be reported as an AE of either hyponatremia or blood sodium decrease. In females, no hyponatremia or blood sodium decrease was reported as an AE throughout the 12‐week treatment period. In males, hyponatremia and blood sodium decrease, as related AEs, were reported by 1.8% and 0.9% of subjects in the 50 μg and 25 μg groups, respectively; all three subjects were ≥65 years of age. There were two AEs of mild intensity, namely hyponatremia in two male subjects receiving 50 μg desmopressin ODT; both these subjects were aged ≥65 years. One of these two subjects was withdrawn from the trial due to the AE; in this subject, serum sodium concentrations were 134 and 133 mM at follow‐up after discontinuation of treatment. The other subject recovered from the AE after the completion of treatment. There was one AE of mild intensity, namely blood sodium decrease, which was reported in one male subject receiving 50 μg desmopressin ODT. This subject was also >65 years of age, and recovered from the AE after the completion of desmopressin administration. The AE of blood sodium decrease was assessed by the investigator to have a reasonable possibility to be related to the desmopressin.

## 4 DISCUSSION

We evaluated the efficacy and safety of desmopressin ODTs versus placebo at doses of 25 and 50 μg in Japanese males, and at a dose of 25 μg in Japanese females with nocturia due to NP. In Japanese males, the change from baseline in the mean number of nocturnal voids through the 12 weeks, as a primary endpoint, decreased significantly compared with placebo at both the 25 and 50 μg doses. In addition, 50 and 25 μg desmopressin ODTs provided statistically significant improvement compared with the placebo in terms of prolonging the time to first awakening to void, nocturnal urine volume indicating the pharmacological action of desmopressin ODT, and other efficacy endpoints.

Weiss et al.^6^ and Sand et al.^7^ conducted two randomized placebo‐controlled studies in males and females with two or more nocturnal voids per night to assess the clinical benefit of desmopressin ODTs in females (25 μg) and males (50 μg) with nocturia due to NP. Both groups found stronger treatment effects, such as decreased nocturnal voids, with desmopressin ODT versus placebo, and the magnitude of differences was indicative of clinical benefits in patients with NP. A greater reduction in the mean number of nocturnal voids was seen with desmopressin ODT in males (TC –0.37 voids) than in females (TC: −0.29 voids). The findings of the present study are consistent with the results reported in the similarly designed global study of male‐only participants 0.37, demonstrating the clinical effect of desmopressin ODT with reproducibility.^16^

In Japanese females, the decrease from baseline in the mean number of nocturnal voids in the 25 μg desmopressin ODT group was numerically greater than that in the placebo group (−1.11 and −0.95, respectively). In addition, 25 μg desmopressin ODT significantly improved secondary efficacy endpoints compared with placebo. A statistically significant difference compared with placebo was not found in the change in the mean number of nocturnal voids throughout the 12‐week treatment period. Weiss et al.^16^ reported that 25 μg desmopressin ODT was statistically superior to placebo in all efficacy endpoints in a global female study.

**Figure 5:**
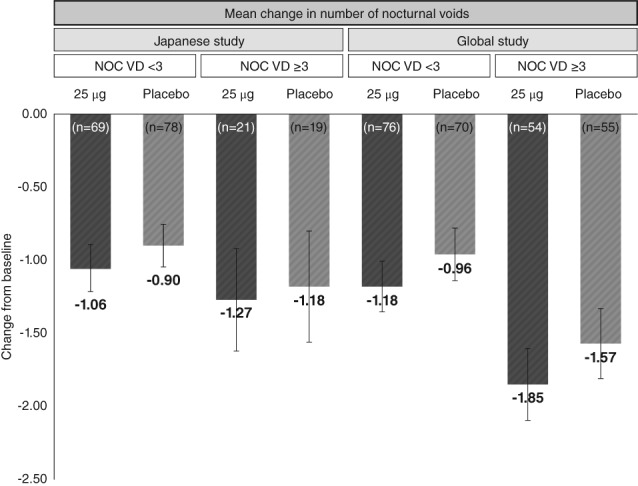
Change from baseline in the mean number of nocturnal voids (NOC VD) stratified by baseline value for females in Japanese (in this present study) versus global studies.^7^ Data are the mean ± 95%CI. ODT, orally dissolving tablet. The numbers below each columns denotes the change from baseline in the mean number of nocturnal voids

The lack of a significant difference in Japanese females in the change in the mean number of nocturnal voids between the 25 μg desmopressin ODT and placebo groups could be due to several reasons. First, an evident placebo effect was seen in females. A numerically greater placebo effect was observed in key efficacy endpoints in females receiving placebo compared with males. This effect was also seen in Japanese patients in the early Phase II study.^8^ This result may not be an effect of the medication in men versus women, but from the high reactions of female patients to placebo. Such reactions to placebo were observed to be higher, depending on the baseline value of nocturnal voids, suggesting possible masking of the medication effects (Figure 5). To avoid the placebo effect we set a 1 week placebo run‐in/lifestyle change period. A longer placebo run‐in period, such as ≥2 weeks, is preferable, such as in recent randomized control trials for lower urinary tract symptoms,^17^ but at this moment there is no clear evidence and general consensus on the optimal length of lifestyle change. In addition, the 1‐week run‐in period allowed the investigator to ascertain which subjects would be most likely to comply with the treatment.

However, it should be noted that the efficacy of 25 μg desmopressin ODT for females has been confirmed as statistically superior to placebo for the change in the mean number of nocturnal voids in global studies.^6^, ^7^ The higher reactions of females in the Japanese study to placebo have also been reported in the development of therapeutic products for patients with irritable bowel syndrome (IBS).^18^, ^19^ In addition, the lifestyle modifications and behavioral reinforcement during the study (through the large number of questionnaires, diaries, and multiple office visits) may have encouraged compliance with lifestyle modifications of females, contributing to the placebo effect. The Nocturia Advisory Conference 2012 of the New England Research Institutes in 2012,^20^ where the panelists reviewed five studies, concluded that “multi‐component behavioral interventions are an attractive approach to a multifactorial condition such as nocturia, despite the relative paucity of supporting data.”

A sex difference in renal sensitivity to desmopressin was confirmed based on the results in previous Japanese and global studies,^8^, ^21^ which affects overall clinical efficacy and safety of the drug. In males and females in the 25 μg group, the mean number of nocturnal voids decreased by −0.96 and −1.11, respectively. The time to first awakening to void was prolonged by 93.37 and 116.11 minutes in males and females, respectively. The findings of this study based on a 25 μg dose desmopressin ODT show that this dose had a numerically higher effect across all efficacy endpoints in females than in males (Tables 3 and 4). This is consistent with the results of global clinical studies, which suggest a sex difference in efficacy.^21^

Sleep disorders caused by waking up at night due to nocturia are known to be involved in decreased work productivity and vitality,^22^ as well as the psychological state and physical factors.^23^, ^24^, ^25^ Therefore, sleep disturbance leads to a lower QoL. Sleep quality is divided into Stages 1 to 4, and Stage 3 and 4 are called deep sleep or slow wave sleep. Stages 3 and 4 commonly occur in the first two cycles (180 minutes) after falling asleep and are considered important for high‐quality sleep.^26^

Furthermore, we investigated the effects of desmopressin ODT on health‐related QoL, sleep quality, and bother using the N‐QoL, ISI, and Hsu five‐point Likert bother scale, respectively, in an exploratory manner. We were not able to show any significant differences, except for the Hsu five‐point Likert bother scale, but the improvements favored 50 and 25 μg desmopressin ODTs versus placebo for all measurements, which substantiates the benefits of desmopressin ODT as a treatment for nocturia. We emphasize that 50 and 25 μg desmopressin ODTs in male patients maintained the time to first awakening to void at >4 hours. It should be the patient benefit as maintain of the first 3 hours in time to first awakening to void at >3 hours is benefit for the patients, which is important for improving QoL.^15^, ^26^, ^27^, ^28^, ^29^ Furthermore, the percentage of sleep responders (ie, the percentage [adjusted value] of subjects who achieved mean time to first awakening to void of at least 180 minutes through the 12 weeks) was 82%, 78%, and 63% in the 50 and 25 μg desmopressin ODT and placebo groups, respectively (odds ratio [vs placebo] 2.68 and 2.07 in the 50 and 25 μg desmopressin ODT groups, respectively). Similar results were found in females treated with 25 μg desmopressin ODT (time to first void at least 180 minutes [sleep responder]: 78%; odds ratio vs placebo 1.67).

**Figure 6:**
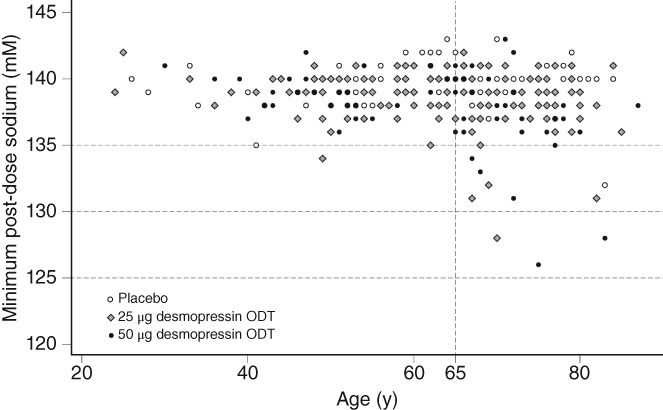
Plot of minimum post‐dosing serum sodium concentrations in male subjects according to age. ODT, orally dissolving tablet

**Figure 7:**
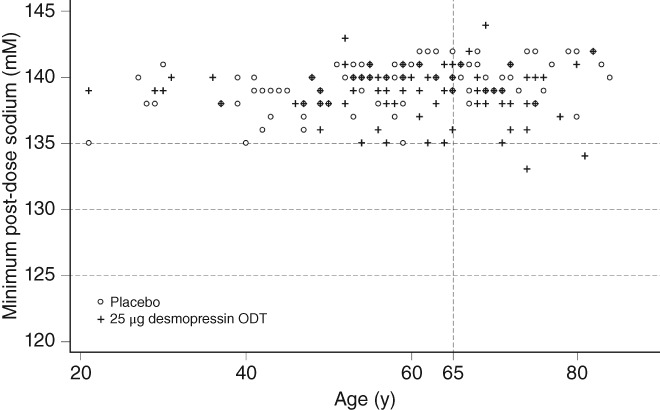
Plot of minimum post‐dosing serum sodium concentrations in female subjects according to age. ODT, orally dissolving tablet

Hyponatremia is considered to be a serum sodium concentration < 135 mM, which is below the standard range (135–145 mM), but the serum sodium concentration associated with clinically significant hyponatremia is <130 mM. In these studies, we found that hyponatremia with a decrease in serum sodium to ≤130 mM occurred in elderly patients (at least 65 years of age) in association with the desmopressin ODT (Figures 6 and 7) in two and one male patient in the 50 and 25 μg, groups, respectively, and there was no severe hyponatremia (defined as a decrease in serum sodium concentrations to ≤125 mM) throughout the 12 weeks. In most subjects with serum sodium concentrations <135 mM, the hyponatremia manifested within the first week of commencing desmopressin. All occurrences of hyponatremia or blood sodium decrease as an AE were asymptomatic and mild, as well as reversible, because sodium concentrations quickly recovered after discontinuation of desmopressin. Furthermore, in the present studies the risk of hyponatremia was lower than in global studies.^6^, ^7^ Moreover, hyponatremia is predictable, often occurring within 1 week of the start of treatment.

It has been suggested that the risk of hyponatremia or serum sodium decrease could be minimized by selecting the appropriate dose according to the patient's background and measurement of serum sodium concentrations,^30^ although the occurrence of hyponatremia could not be ruled out because of the pharmacological action of desmopressin.^30^ No other events indicating safety concerns were observed, confirming that the 25 and 50 μg desmopressin ODTs are safe and well tolerated.

In male patients with nocturia due to NP who were treated with 50 and 25 μg desmopressin ODTs throughout the 12 weeks, the primary objective was achieved and efficacy was consistently observed in time to first awakening to void, nocturnal urine volume, and NPI as secondary endpoints. These results indicate the clinical benefits of desmopressin ODT that include not only a reduction in nocturnal voids, but also an effective prolongation in the time to first awakening to void, which had the greatest effect on improving sleep quality, and represented the true aim of nocturia treatment. The effects of desmopressin ODT were statistically significant. Furthermore, the treatment improved health‐related QoL in nocturia patients. In males, 50 μg desmopressin had higher efficacy than did 25 μg desmopressin and there were no safety issues.

The findings above confirm the superiority of 50 and 25 μg desmopressin ODTs compared with placebo for Japanese males. Therefore, we determined that 50 μg desmopressin ODT is an appropriate dose for males with nocturia due to NP. For patients who are more likely to experience hyponatremia, especially elderly male patients with low body weight (<50 kg), low body mass index (<18.5 kg/m^2^), poor physiological status, or impaired renal function, 25 μg desmopressin ODT should be considered as a starting dose and the patients should be frequently monitored for hyponatremia.

In summary, the results of this study demonstrate that 50 and 25 μg desmopressin ODTs were efficacious in treating Japanese male patients with nocturia due to NP. In addition, 50 and 25 μg desmopressin ODTs were determined to be safe and well tolerated; therefore, it can be concluded that the risk–benefit profiles for 50 and 25 μg desmopressin ODTs are favorable for treating male patients with nocturia due to NP. In particular, the findings also determined that 50 μg desmopressin ODT is the optimal dose for males, providing clinically meaningful benefits. For elderly patients who are more likely to experience hyponatremia, 25 μg desmopressin ODT should be considered as a starting dose. With regard to Japanese female patients with nocturia due to NP, 25 μg desmopressin ODT once daily decreased the number of nocturnal voids, but the superiority of 25 μg desmopressin ODT over placebo could not be confirmed statistically, although global clinical trials have shown significant nocturnal void reduction in females.^7^

## DISCLOSURE

Osamu Yamaguchi is a consultant to Ferring Pharmaceuticals. Kristian Vinter Juul, Ali Falahati, Toru Yoshimura, Futoshi Imura, and Mikiya Kitamura are employees of Ferring Pharmaceuticals.

## APPENDIX 1 1

List of principal investigators:

Masashige Yoshida, Ogikubo Ekimae Clinic; Toru Tanaka, Toru Clinic; Hajime Ogawa, Ogawa Clinic; Keiichi Miyakoda, Miyakoda Urological Clinic; Fumikazu Yuge, Yuge Clinic; Kiyoshi Nakamura, Kunitachi Sakura Hospital; Tadashi Uemura, Uemura Clinic; Kenji Harada, Harada Urology Clinic; Tetsuo Yamaguchi, Yamaguchi Clinic; Hiroshi Fukuda, Fukuda Urological Clinic; Kazuhide Kondo, Kondo Clinic; Yukio Tsujimoto, Tsujimoto Urological Clinic; Mototaka Hayashi, Hayashi dermatology/urology/internal medicine Clinic; Shuso Den, Den Urology Clinic; Hiroshi Kameoka, Kameoka Clinic; Tomokazu Umeyama, Umeyama Clinic; Yuichi Kato, Kato Clinic; Masaki Yamanaka, Yamanaka Clinic; Kaoru Nakamura, Nakamura Urology Clinic; Makoto Suzuki, Ocean Clinic; Hidenori Sumiya, Sumiya Clinic; Hironao Itakura, Kusunoki Clinic; Atsushi Iwasa, Iwasa Clinic; Masayuki Sugimoto, Koganeibashi Sakura Clinic; Kosaku Yasuda, Yasuda Urology Clinic; Hiroyuki Kusuyama, Eikou Clinic; Akihito Akiyama, Shibuya Shinminamiguchi Clinic; Fumio Manabe, Touyu Clinic; Toshiyuki Kamijo, Kamijo Clinic; Takashi Kuwabara, Tokyo Kamata Hospital; Takashi Nakanoma, Nakanoma Clinic Urology Department; Hiroshi Ohkusa, Ookusa Urology & Internal Medicine Clinic; Hiromasa Kiura, Kiura Urological Clinic; Hideya Kuroda, Kuroda Urological Clinic; Tohru Hirata, Hirata Internal Medicine Urology Clinic; Shuichi Nakagawa, Nakagawa Clinic; Takahiro Sakuma, Sakuma Clinic; Yoshihiro Nakamura, Urology Nakamura Clinic; Kunio Maruyama, Taisei Clinic; Masahiro Ishihara, Tachikawa Kitaguchi Ekimae Clinic; Nobuaki Furugen, Shintoshin Clinic; Toru Hihara, Hihara Clinic; Teruo Yajima, Yajima Surgery Urological Clinic; Tadashi Kotake, Clinic Tsudanuma; Hiroyuki Shimizu, Hasegawa Hospital; Ryuichi Saito, Ryokuen Saito Clinic; Eisuke Iwasa, Iwasa Urological Clinic; Isao Hirano, Hirano Clinic; Shuichi Hida, Hida Urological Clinic; Takahide Hosobe, Hosobe Clinic; Noriyuki Otani, Obihiro Urological Clinic; Kazuaki Takahashi, Nishi2jyo Jin Urology Hospital; Toshihiko Saito, Chiba Central Medical Center; Shinji Iwata, Iwata Clinic; Shigeto Araki, Araki Clinic; Toshihiko Yamazaki, Yamazaki Surgical Urological Clinic; Keisuke Yamamoto, Yamamoto Clinic.
